# C-reactive protein-to-albumin ratio predicts intensive care admission and disease severity in autoimmune encephalitis

**DOI:** 10.3389/fimmu.2026.1699620

**Published:** 2026-02-02

**Authors:** Lin-Jie Zhang, Zewen Han, Ying-Zhe Shao, Qiu-Xia Zhang, Ning Zhao, Xiao-Yi Xu, Li Yang

**Affiliations:** 1 Department of Neurology, Tianjin Neurological Institute, Tianjin Medical University General Hospital, Tianjin, China; 2 Shenzhen Clinical College of Stomatology, School of Stomatology, Southern Medical University, Shenzhen, Guangdong, China; 3 Shenzhen Stomatology Hospital (Pingshan) of Southern Medical University, Shenzhen, Guangdong, China

**Keywords:** autoimmune encephalitis, C-reactive protein/albumin ratio, ICU admission, NMDAR encephalitis, prognostic biomarker

## Abstract

**Objective:**

To evaluate the clinical relevance of the C-reactive protein/albumin ratio (CAR) in patients with autoimmune encephalitis (AE), with an emphasis on its predictive utility for disease severity, intensive care unit (ICU) admission, and functional outcomes.

**Methods:**

A retrospective cohort of 114 patients with AE was analyzed. Serum C-reactive protein (CRP) and albumin (ALB) levels were measured within 24 hours of admission, and CAR was subsequently calculated. Disease severity was assessed using the Clinical Assessment Scale for Autoimmune Encephalitis (CASE) and the modified Rankin Scale (mRS) at discharge. Statistical analyses included the Mann–Whitney U test, Spearman correlation, logistic regression, and receiver operating characteristic (ROC) curve analysis to evaluate associations with ICU admission, respiratory failure, and disability.

**Results:**

Patients requiring ICU admission exhibited significantly elevated CRP levels (11.00 vs. 2.40 mg/L, p < 0.001), reduced ALB levels (36.00 vs. 38.00 g/L, p = 0.029), and higher CAR values (0.282 vs. 0.064, p < 0.001). Comparable patterns were observed in patients with respiratory failure and severe disability (mRS ≥ 3). CAR demonstrated stronger correlations with both CASE score at admission (r = 0.448, p < 0.001) and mRS at discharge (r = 0.222, p = 0.018) than either CRP or ALB alone. Multivariate logistic regression analysis, adjusted for age, sex, CASE score, and other potential confounders, identified CAR (OR = 2.100; 95% CI: 1.151–3.831; p = 0.016), CRP (OR = 1.023; 95% CI: 1.004–1.042; p = 0.015), and ALB (OR = 0.875; 95% CI: 0.787–0.973; p = 0.013) as independent predictors of ICU admission. ROC curve analysis indicated high predictive accuracy for CAR (AUC = 0.835; cutoff = 0.125; sensitivity = 91.3%) and CRP (AUC = 0.820; cutoff = 4.35; sensitivity = 82.6%).

**Conclusion:**

CAR represents a novel and readily accessible biomarker that outperforms CRP or ALB alone in predicting disease severity and the need for ICU care in patients with AE. Its incorporation into early clinical assessment protocols may enhance risk stratification and inform decisions regarding intensive care resource allocation.

## 1 Introduction

Autoimmune encephalitis (AE) comprises a heterogeneous group of neuroinflammatory disorders resulting from immune-mediated attacks on central nervous system antigens, presenting with a range of clinical manifestations including seizures, cognitive dysfunction, and psychiatric disturbances (1). The early identification of patients at risk for severe disease progression or requiring intensive care unit (ICU) admission remains a major challenge in clinical settings. Although antibody testing and neuroimaging facilitate diagnosis, there remains an urgent need for readily available biomarkers capable of predicting disease severity and guiding therapeutic decision-making (2).

The C-reactive protein/albumin ratio (CAR), a composite index reflecting both inflammation and nutritional status, has been proposed as a prognostic biomarker in systemic inflammatory conditions such as sepsis and malignancy (3, 4). C-reactive protein (CRP), an acute-phase reactant, reflects systemic inflammatory responses, whereas albumin (ALB) levels are inversely associated with disease severity due to their anti-inflammatory properties and role in maintaining endothelial integrity (4–8). CAR integrates these opposing physiological dynamics, thereby offering potentially superior prognostic value in acute clinical contexts. Recent studies have demonstrated that CAR serves as a prognostic indicator in various neurological disorders, including stroke and traumatic brain injury (9, 10). An observational study reported that patients with acute ischemic stroke who presented with elevated CAR levels at admission were more likely to develop stroke-associated pneumonia during hospitalization (11). Additional evidence indicates that critically ill patients undergoing surgery under general anesthesia with mechanical ventilation exhibit significantly elevated CAR values compared to those not requiring intubation (12). Elevated CAR reflects a synergistic combination of heightened inflammation and impaired nutritional status, which may offer enhanced prognostic relevance in AE. Nevertheless, its clinical applicability in AE, a disorder whose pathogenesis is rooted in neuroinflammatory processes, has not been systematically investigated.

The present study investigates the clinical significance of CAR in a cohort of 114 patients diagnosed with AE. It is hypothesized that an elevated CAR at the time of admission correlates with disease severity and serves as a predictor of ICU admission. By comparing the predictive performance of CAR with CRP and ALB individually, the study aims to validate its superiority as a biomarker for risk stratification and to support its integration into early clinical decision-making frameworks.

## 2 Subjects and methods

### 2.1 Patients

We retrospectively analyzed the data of 114 patients with autoimmune encephalitis (AE) who were registered and enrolled at the Department of Neurology, Tianjin Medical University General Hospital between August 2013 and April 2025. Inclusion criteria were as follows (1): age ≥16 years (2); diagnosis of AE in accordance with established consensus criteria (1); (3) detection of definitive AE-associated antibodies in serum and/or cerebrospinal fluid (CSF); (4) availability of complete clinical records. The exclusion criteria included: (1) concurrent acute neurological disorders; (2) history of pre-existing movement disorders; (3) significant chronic comorbidities, including hematological, renal, or hepatic diseases; and (4) receipt of immunotherapy within 4 weeks prior to admission, such as corticosteroids, steroid-sparing agents, intravenous immunoglobulin (IVIG), plasma exchange, or biological agents, for expmple rituximab or cyclophosphamide; (5) Presence of definite systemic bacterial or fungal infection at admission. The study was conducted in accordance with the ethical principles of the Declaration of Helsinki and received approval from the Ethics Committee of Tianjin Medical University General Hospital (IRB2025-YX-308-01).

### 2.2 Data collection

Comprehensive clinical data were systematically retrieved from electronic medical records, including demographic variables (age at onset, sex), primary clinical manifestations, and outcome metrics. Disease severity was assessed using two validated clinical instruments: (1) the modified Rankin Scale (mRS) at discharge, ranging from 0 (no symptoms) to 6 (death), and (2) the Clinical Assessment Scale for Autoimmune Encephalitis (CASE) (13), which evaluates nine symptom domains (seizures, memory dysfunction, psychiatric symptoms, impaired consciousness, language deficits, dyskinesia, gait instability or ataxia, brainstem dysfunction, and motor weakness), yielding a total score ranging from 0 to 27, assessing the acute severity of various clinical symptoms.

Laboratory parameters included serum C-reactive protein (CRP) and albumin (ALB) levels measured within 24 hours of admission. Data were extracted following the hospital’s unified standardized electronic medical record system. Laboratory measurements included CRP, assessed by immunonephelometry, and ALB, determined by the bromocresol green method, with both assays undergoing continuous standardized quality control. The C-reactive protein-to-albumin ratio (CAR) was derived by dividing the serum CRP level (mg/L) by the albumin level (g/L), and the result was then multiplied by 10. Serum and/or cerebrospinal fluid (CSF) samples were subjected to standardized antibody testing using indirect immunofluorescence assays. All enrolled patients had CRP and ALB test results available after admission within 24 hours, with no missing data.

Patients were categorized into ICU and non-ICU groups based on admission status. ICU admission was based on the presence of one or more of the following criteria: (1) life-threatening neurological complications (e.g., status epilepticus, coma, or severe delirium); (2) respiratory failure necessitating mechanical ventilation; (3) hemodynamic instability requiring vasopressor therapy; (4) severe sepsis or multiorgan dysfunction; (5) elevated intracranial pressure warranting intervention; or (6) an Acute Physiology and Chronic Health Evaluation II (APACHE II) score indicative of critical illness. In accordance with established critical care research standards, only patients with ICU stays lasting ≥48 hours were included in the ICU cohort.

### 2.3 Statistical analysis

Continuous variables were analyzed based on their distribution characteristics. Variables with a normal distribution, verified by normality testing, were expressed as mean ± standard deviation and compared using the independent-samples Student’s t-test. Non-normally distributed data were reported as median and interquartile range (IQR), and comparisons were performed using the Mann-Whitney U test. Spearman correlation was used to assess the relationship between inflammatory markers (CRP, ALB, CAR) and disease severity scores. The independent predictive value of inflammatory markers at admission for clinical outcomes was evaluated using multivariate logistic regression, with odds ratios (ORs) and corresponding 95% confidence intervals (CIs) calculated. The discriminative performance of CRP, ALB, and CAR in predicting ICU admission and respiratory failure was assessed using receiver operating characteristic (ROC) curve analysis. The area under the curve (AUC) and 95% CIs were reported for each marker, with optimal cutoff values determined using Youden’s index. Given the imbalance in group sizes, multivariate logistic regression models were used to adjust for potential confounders, rather than propensity score matching. All statistical tests were two-tailed, and a p-value < 0.05 was considered indicative of statistical significance. All statistical analyses were conducted using IBM SPSS Statistics (version 26; IBM Corp., Armonk, NY). Graphical visualizations were generated using GraphPad Prism (version 6.01; GraphPad Software, San Diego, CA).

## 3 Results

### 3.1 Demographic and clinical characteristics

**Figure 1:**
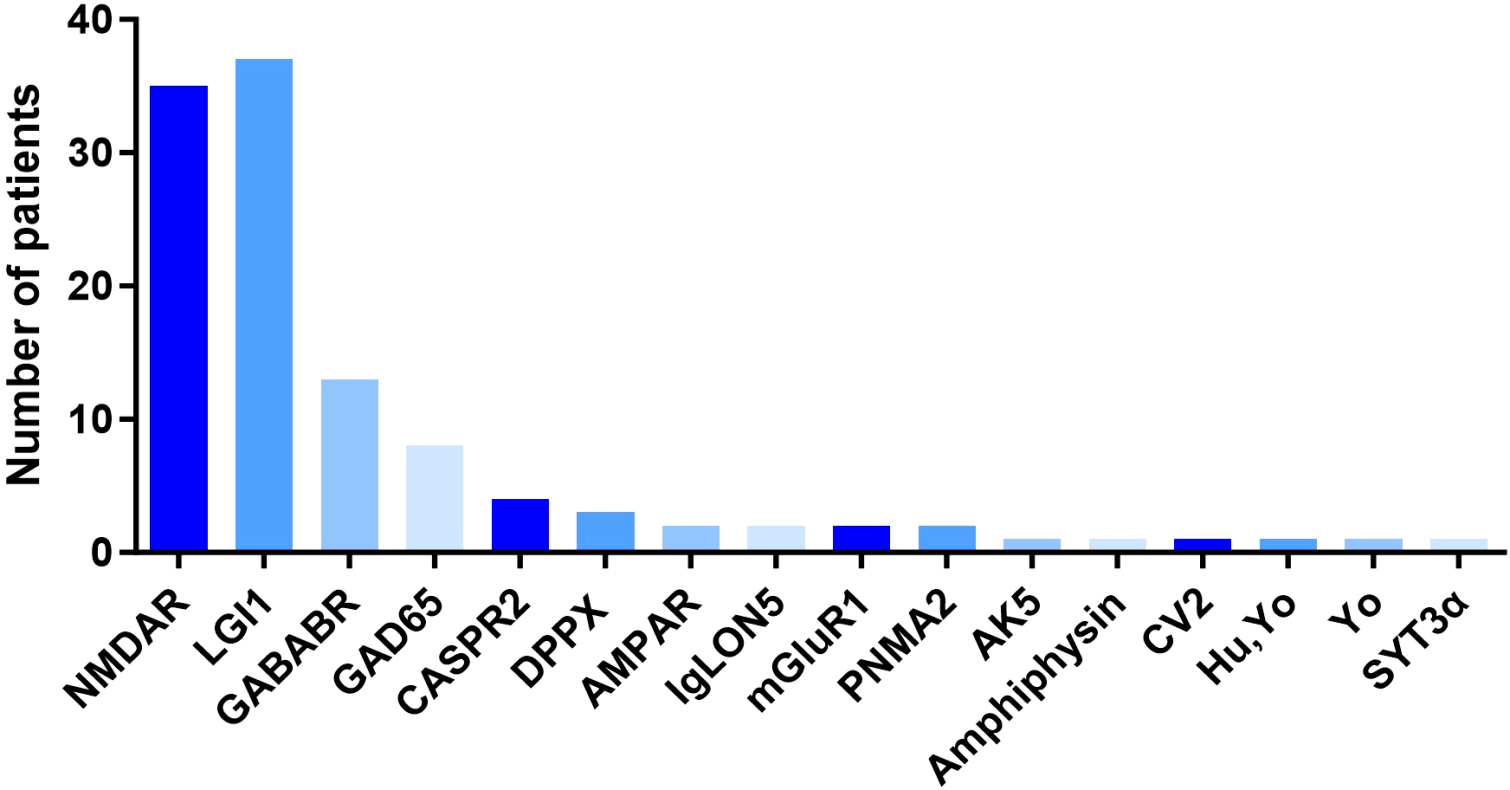
Number of patients with different autoantibodies in AE. NMDAR, Anti-N-methyl-D-aspartate receptor; LGI-1, Leucine-rich glioma inactivated 1; GABA, G-aminobutyric acid type; GAD65, Glutamic acid decarboxylase-65; CASPR2, Contactin associated protein 2; DPPX, Dipeptidyl-peptiddase-like protein-6; AMPAR, Amino-3-hydroxy-5-hydroxy-5-methyl-4-isoxazolepropionic acid receptor; IgLON5, Immunoglobulin-like domain-containing neuronal protein 5; mGluR1, Metabotropic Glutamate Receptor 1; PNMA2, Paraneoplastic Ma Antigen 2; AK5, Adenylate Kinase 5; CV2, Collapsin Response Mediator Protein 5; SYT3α, Synaptotagmin-3α.

A total of 114 patients diagnosed with autoimmune encephalitis (AE) were retrospectively enrolled between August 2013 and April 2025. With a mean age at onset of 50.63 ± 18.90 years, the cohort consisted of 74 males (64.91%) and 40 females (35.09%), demonstrating a male predominance. Antibody profiling identified anti-leucine-rich glioma-inactivated 1 (LGI1) antibodies as the most prevalent subtype (32.46%, n = 37), followed by anti-N-methyl-D-aspartate receptor (NMDAR) (30.70%, n = 35), anti-γ-aminobutyric acid B receptor (GABABR) (11.40%, n = 13), and anti-glutamic acid decarboxylase 65 (GAD65) (7.02%, n = 8). The remaining patients were positive for other AE-associated antibodies (18.42%, n = 21) (**Figure 1**). Disease severity at admission was assessed using the Clinical Assessment Scale for Autoimmune Encephalitis (CASE), with a median score of 5.00 (IQR: 7.25), indicating moderate severity. The median modified Rankin Scale (mRS) score at admiddion was 3.00 (IQR: 3.00). At discharge, the median mRS score improved to 2.00 (IQR: 2.00), reflecting generally favorable short-term recovery, while 31.60% (n = 36) of patients exhibited significant disability (mRS ≥ 3). During hospitalization, 23 patients (20.20%) required intensive care unit (ICU) admission. and 14 (12.30%) developed respiratory failure. Initial immunotherapy regimens included intravenous methylprednisolone (IVMP) monotherapy in 44.74% (n = 51), IVMP combined with intravenous immunoglobulin (IVIG) in another 44.74% (n = 51), and IVIG monotherapy in 5.26% (n = 6). Two critically ill patients were treated with IVMP combined with IVIG, and required adjunctive double-filtration plasmapheresis (DFPP) due to a lack of improvement. Four patients did not receive immunotherapy. Second-line immunotherapy with rituximab (RTX) was administered in 16.66% (n = 19), including 6 cases (5.26%) who also received daratumumab (DARA). In addition, 5 patients (4.39%) underwent targeted pathogenic antibody clearance using efgartigimod.

### 3.2 Association between inflammatory markers and disease severity

**Figure 2:**
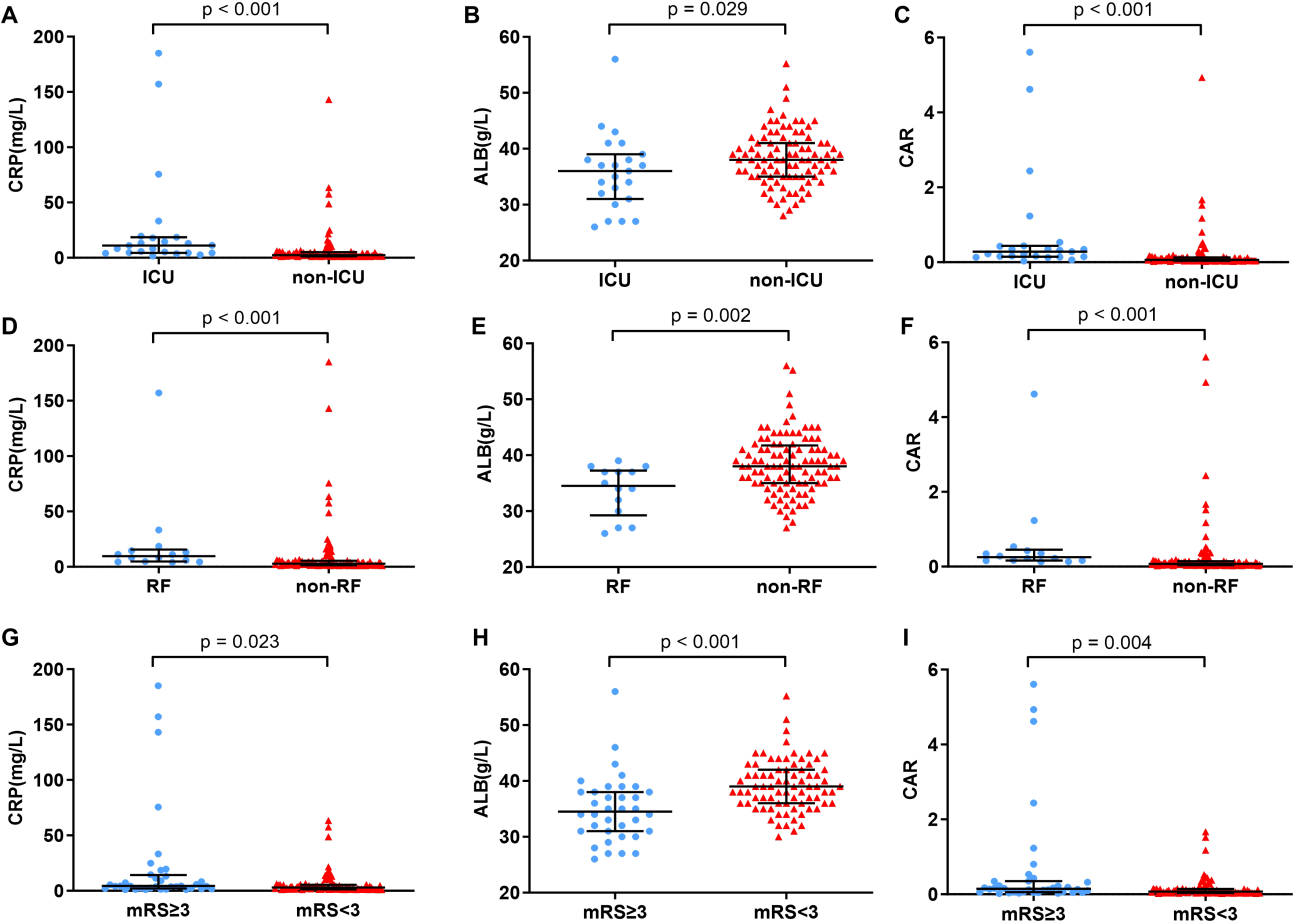
Comparison of CRP, ALB and CAR between AE patients with different severity. **(A–C)** Comparison of CRP, ALB and CAR between AE patients with ICU admission and non-ICU admission; **(D–F)** Comparison of CRP, ALB and CAR between AE patients with respiratory failure and non-respiratory failure; **(G–I)** Comparison of CRP, ALB and CAR between AE patients with discharge mRS≥3 and discharge mRS<3.

Specifically, the median CRP level was 3.60 mg/L (IQR: 5.30), the median albumin level was 38.00 g/L (IQR: 6.25), and the median CAR was 0.097 (IQR: 0.139). Significant differences in inflammatory markers were observed across clinical severity subgroups (**Figure 2**). Patients requiring ICU admission had markedly elevated serum CRP levels (median 11.00 vs. 2.40 mg/L, p < 0.001), decreased albumin concentrations (36.00 vs. 38.00 g/L, p = 0.029), and consequently higher CAR values (0.282 vs. 0.064, p < 0.001) compared to non-ICU patients. A similar pattern was noted among patients with respiratory failure, who exhibited elevated CRP (9.60 vs. 2.70 mg/L, p < 0.001), more pronounced hypoalbuminemia (34.50 vs. 38.00 g/L, p = 0.002), and higher CAR (0.252 vs. 0.070, p < 0.001). Furthermore, CAR values were significantly elevated in patients with poor functional outcomes (mRS ≥ 3), who demonstrated higher CRP (4.35 vs. 2.90 mg/L, p = 0.023), lower albumin levels (34.50 vs. 39.00 g/L, p < 0.001), and increased CAR (0.147 vs. 0.073, p = 0.004) relative to those with favorable outcomes.

**Figure 3:**
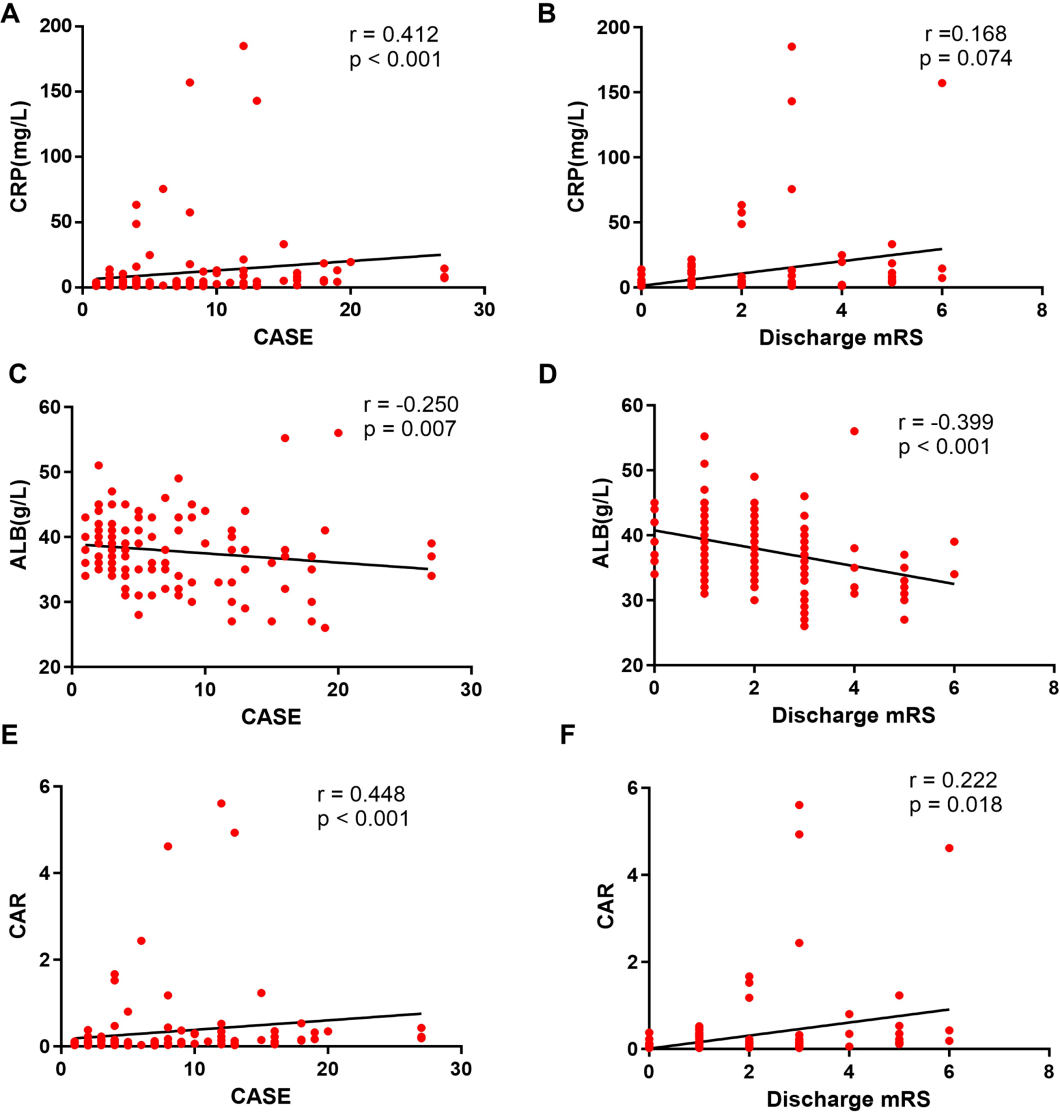
Correlations of CRP, ALB and CAR with severity of AE. **(A, B)** The correlations of CRP with CASE score and discharge mRS score were assessed using Spearman correlation analysis. **(C, D)** The correlations of ALB with CASE score and discharge mRS score were assessed using Spearman correlation analysis. **(E, F)** The correlations of CAR with CASE score and discharge mRS score were assessed using Spearman correlation analysis.

Spearman correlation analyses further clarified these associations (**Figure 3**). Both CRP (r = 0.412, p < 0.001) and CAR (r = 0.448, p < 0.001) showed strong positive correlations with CASE scores at admission. In addition, CAR demonstrated a modest but significant correlation with mRS scores at discharge (r = 0.222, p = 0.018). Conversely, albumin levels were inversely correlated with both CASE (r = –0.250, p = 0.007) and mRS scores (r = –0.399, p < 0.001), underscoring the dual prognostic role of inflammatory and nutritional status.

### 3.3 CRP, ALB, and CAR levels across clinical subgroups

**Figure 4:**
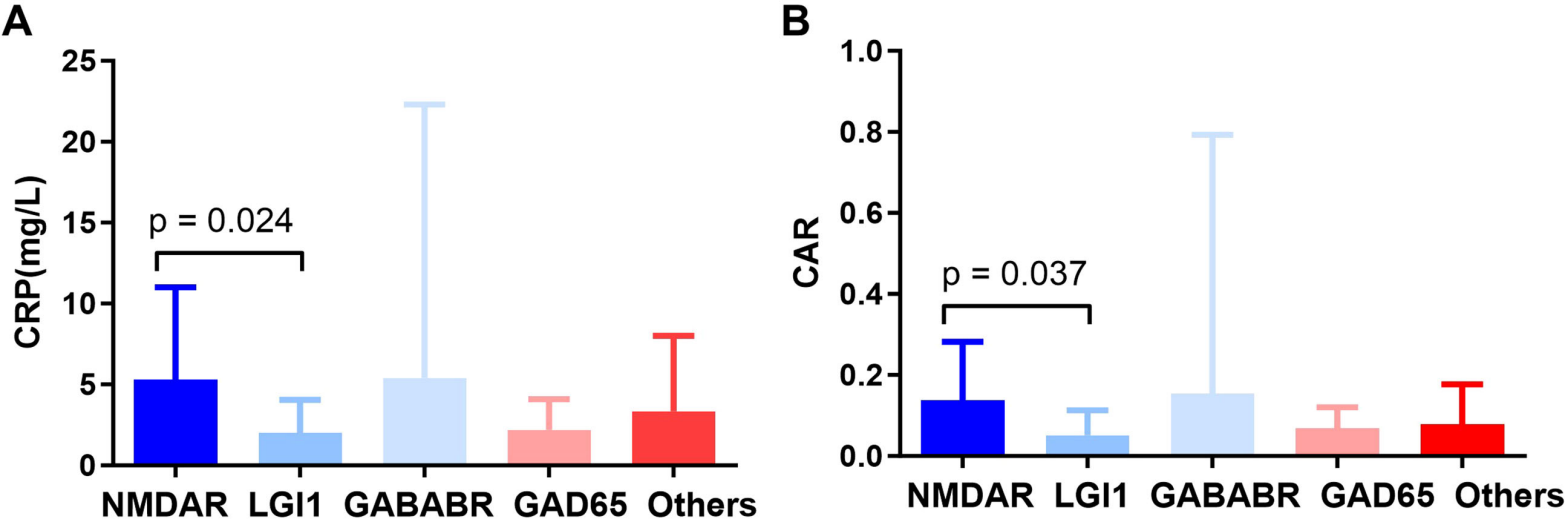
CRP and CAR levels across different antibody-defined subgroups. **(A)** CRP levels across different antibody-defined subgroups. **(B)** CAR across different antibody-defined subgroups.

We compared CRP, ALB, and CAR levels across different antibody-defined subgroups, considering individual groups for antibodies with >5 cases and consolidating rarer types into an “Other” category. The Kruskal–Wallis test indicated significant overall differences for CRP (p=0.011) and CAR (p=0.014) but not for ALB (p=0.500). *Post-hoc* pairwise comparisons with Bonferroni correction revealed that the NMDAR subgroup had significantly higher CRP (p=0.024) and CAR (p=0.037) than the LGI1 subgroup, while no other inter-group differences reached significance (all p>0.05) (**Figure 4**). Furthermore, no significant differences in CRP, ALB, or CAR were observed between patients with and without paraneoplastic AE (all p>0.05).

### 3.4 Predictive value of inflammatory markers for ICU admission

**Figure 5:**
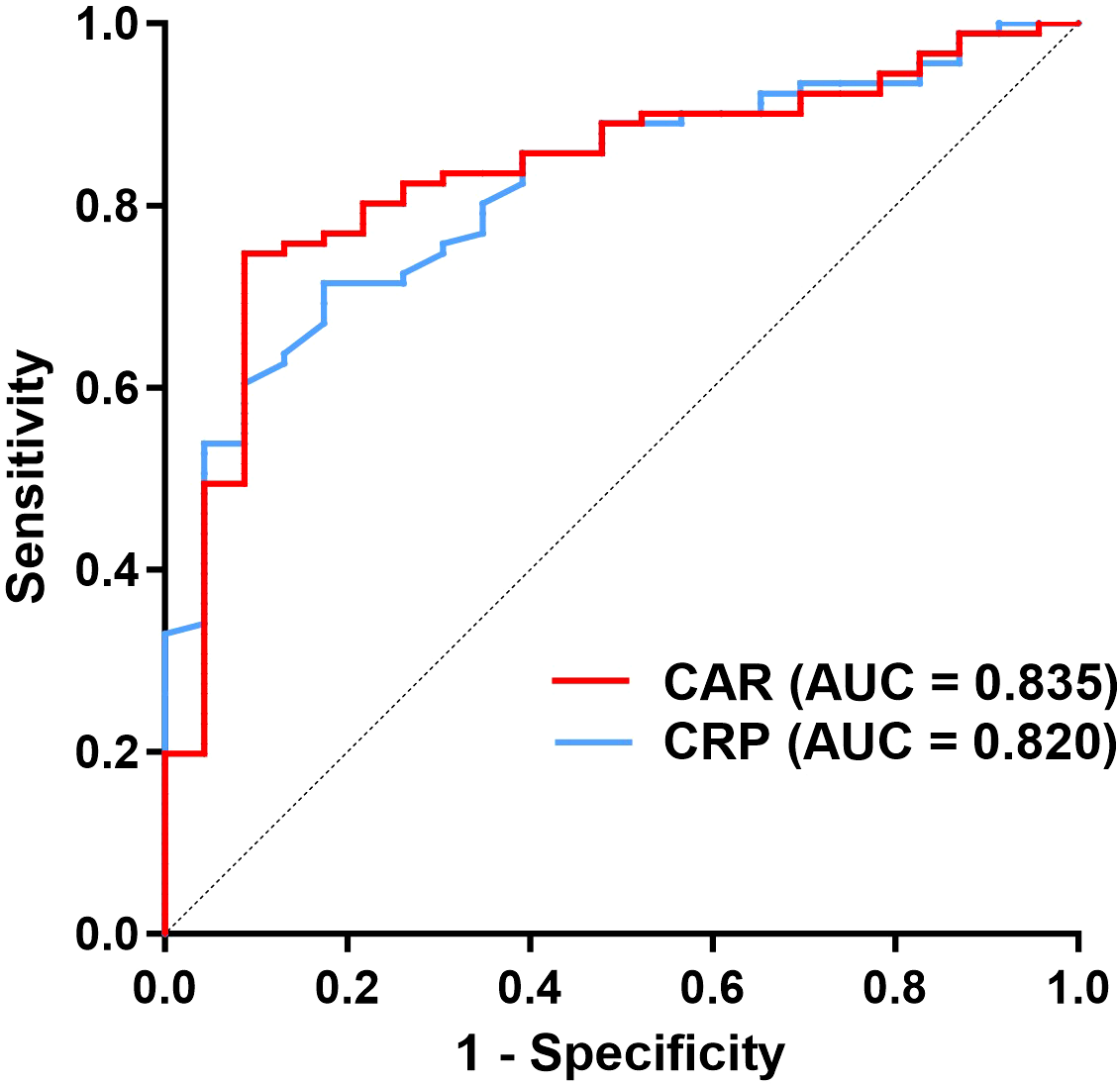
ROC curves of the CRP and CAR on predicting ICU admission in AE patients. ROC, receiver operating characteristic; ICU, intensive care unit; AE, autoimmune encephalitis; CRP, C-reactive protein; CAR, CRP to albumin ratio.

Multivariate logistic regression, adjusted for age, sex, CASE scores, positive neuronal surface antigen antibodies and onset-to-admission interval (days) identified CRP (OR = 1.023; 95% CI: 1.004–1.042; p = 0.015), ALB (OR = 0.875; 95% CI: 0.787–0.973; p = 0.013) and CAR (OR = 2.100; 95% CI: 1.151–3.831; p = 0.016) as independent predictors of ICU admission (**Table 1**). Although ALB (OR = 0.739; 95% CI: 0.620–0.880; p = 0.001) has predictive value for respiratory failure, neither CRP nor CAR shows statistically significant differences (p =0.085; p = 0.064). Receiver operating characteristic (ROC) analysis confirmed the strong discriminative power of CAR (AUC = 0.835; 95% CI: 0.748–0.922, p < 0.001) and CRP (AUC = 0.820; 95% CI: 0.735–0.904, p < 0.001) for ICU admission prediction (**Figure 5**). The optimal CAR threshold of 0.125 yielded a sensitivity of 91.3% and specificity of 74.7%, outperforming CRP’s optimal cutoff of 4.35 mg/L (sensitivity: 82.6%; specificity: 71.4%). These findings highlight the utility of CAR as a composite biomarker integrating inflammatory burden and nutritional status, offering superior predictive performance for ICU need compared to its individual components.

**Table 1:** Logistic regression of CRP, ALB and CAR on ICU admission in AE.

Variables	CRP			ALB			CAR		
	OR	95%CI	*p*-Value	OR	95%CI	*p*-Value	OR	95%CI	*p*-Value
Age	0.977	0.951-1.004	0.088	0.971	0.943-0.999	0.045	0.976	0.950-1.003	0.081
Sex	0.798	0.276-2.308	0.677	0.973	0.327-2.893	0.960	0.779	0.270-2.249	0.644
CASE	1.011	0.930-1.098	0.798	1.021	0.939-1.109	0.629	1.011	0.930-1.098	0.798
Positive neuronal surface antigen antibodies	0.825	0.181-3.770	0.804	0.966	0.225-4.154	0.963	0.803	0.172-3.748	0.780
Onset-to-admission interval (days)	0.990	0.978-1.003	0.144	0.991	0.979-1.004	0.159	0.990	0.978-1.003	0.140
CRP	1.023	1.004-1.042	0.015	-	-	-	-	-	–
ALB	-	-	-	0.875	0.787-0.973	0.013	-	-	–
CAR	-	-	-	-	-	-	2.100	1.151-3.831	0.016

## 4 Discussion

Severe AE is a life-threatening condition often requiring ICU admission and airway support due to complications. Early risk stratification is critical for timely intervention. This study identifies the CAR as a reliable and clinically meaningful prognostic biomarker in AE. Our findings demonstrate that elevated CAR values at admission are significantly associated with both acute disease severity and the need for ICU admission. These results suggest that CAR may serve as an accessible and cost-effective tool for early risk stratification in AE management.

Compared with individual measurements of CRP or albumin, CAR exhibited superior predictive performance. This enhanced utility likely reflects its integration of two distinct yet complementary pathophysiological processes: systemic inflammation and nutritional/metabolic status. The strong correlations between CAR and admission CASE score and discharge mRS scores highlight its relevance to both early disease burden and functional outcomes. These results are consistent with previous studies that have reported CAR as a prognostic marker in other neuroinflammatory conditions, including Guillain-Barré syndrome (5).

Importantly, hypoalbuminemia alone was inversely associated with both CASE and mRS scores, further supporting its role as a surrogate for systemic inflammation and blood-brain barrier (BBB) dysfunction. Albumin reduction has been observed in other neuroimmunological disorders, such as Guillain-Barré syndrome, particularly following immune activation or IVIG treatment (14). In AE, low albumin may also reflect increased vascular permeability driven by cytokine-mediated endothelial injury. To further contextualize the predictive value of CAR, we explored its behavior across key clinical subgroups.

Beyond the pooled analysis, we conducted preliminary explorations to assess the influence of clinically relevant factors. Subgroup analysis indicated that patients with NMDAR-AE tended to exhibit higher CRP and CAR compared to those with LGI1-AE. This observation suggests that CAR may vary across AE entities with distinct immunopathogenic mechanisms, warranting future investigation in larger, subtype-homogeneous cohorts. Furthermore, our expanded multivariate model indicated that the predictive value of CAR for ICU admission remained significant even after adjusting for positive neuronal surface antigen antibodies, onset-to-admission interval (days) together with age, sex, and CASE score, underscoring its robustness as a biomarker. CAR outperformed both CRP and albumin individually, with an area under the ROC curve (AUC) of 0.835 and an optimal cutoff value of 0.125 yielding high sensitivity (91.3%) and specificity (74.7%). This finding is clinically significant, as it provides an early, objective measure for identifying patients at high risk for deterioration who may benefit from escalated monitoring or early initiation of second-line immunotherapy. These results build on existing literature supporting the role of CAR as a prognostic tool in critical care populations (15), as well as in neurocritical illnesses such as traumatic brain injury (9) and GBS-associated respiratory failure (5), its application in AE represents a novel extension. The distinct contribution of this study lies in being the first to systematically validate CAR as a predictive biomarker specifically in AE—a disorder primarily driven by CNS-specific autoimmunity—and to link it decisively to the critical endpoint of ICU admission. This positions CAR not merely as another inflammatory ratio but as a practical, accessible tool for early risk stratification in AE, potentially guiding monitoring intensity and resource allocation decisions at a pivotal point in patient management. This study focused on antibody-positive AE patients to ensure diagnostic certainty and to lay the groundwork for future exploration of CAR differences across antibody subtypes, particularly given the distinct pathogenic mechanisms associated with antibodies targeting cell-surface versus intracellular antigens in autoimmune encephalitis.

The underlying mechanisms that confer predictive value to CAR in AE are likely multifactorial. Elevated CRP reflects systemic inflammation, which may parallel central nervous system (CNS) immune activation. Concurrently, hypoalbuminemia may signal malnutrition, hepatic dysfunction, or inflammatory-mediated protein leakage across a compromised BBB (16). In the specific context of AE, this hypoalbuminemia may not merely be a passive nutritional marker but could reflect a state of systemic metabolic dysregulation fueled by the hyperinflammatory response. The intense CNS immune activation characteristic of AE, involving cytokine storms and adaptive immune cell infiltration, is known to have systemic repercussions, potentially altering hepatic synthesis of acute-phase proteins, like CRP and negative-phase proteins, like albumin. The CAR, therefore, represents a composite marker capturing the complex interplay between neuroinflammation-driven systemic immune activation, secondary metabolic stress, and endothelial dysfunction, all of which contribute to disease progression in AE (1). This interpretation is supported by broader evidence demonstrating that CAR reflects the severity of immune-mediated organ dysfunction in autoimmune diseases (17). Furthermore, the preliminary variation in CAR observed across antibody subtypes invites speculation that differences in primary antigen location, inflammatory cytokine profiles, or intensity of systemic involvement among AE subtypes might differentially influence this systemic biomarker ratio, a hypothesis demanding future mechanistic investigation.

From a clinical standpoint, the application of CAR offers several advantages. It is easily calculable using routine laboratory parameters, requires no specialized equipment or assays, and is available in both high-resource and resource-limited settings. The ability to rapidly identify high-risk patients at presentation may inform early therapeutic decisions, including the intensity of immunotherapy, need for ICU transfer, or implementation of additional monitoring strategies. Although our study focused on baseline measurements, future studies should evaluate the utility of serial CAR assessments for monitoring treatment response and guiding therapeutic adjustments. Previous research in GBS has shown that temporal CAR trends mirror clinical improvement or deterioration, suggesting potential value in dynamic monitoring (5).

Several limitations should be acknowledged. This was a single-center study, and external validation in larger, multicenter cohorts is necessary to confirm the generalizability of our findings. Additionally, although we excluded patients with known active infections and found no significant CAR difference in the paraneoplastic subgroup, CAR as a systemic marker could still be influenced by unrecognized subclinical conditions or comorbidities, a limitation inherent to its clinical application. This study analyzed only baseline CAR values at admission, future studies should incorporate longitudinal dynamic monitoring of CAR to assess its relationship with disease activity and treatment response. The mechanistic link between CAR elevation and CNS pathology also warrants further investigation, including correlation with cerebrospinal fluid biomarkers and neuroimaging features.

In conclusion, our study supports the use of CAR as a practical, prognostically informative biomarker in AE. Its strong association with disease severity, functional outcomes, and ICU admission—combined with its accessibility and ease of use—makes it a valuable addition to clinical decision-making in AE. Future research should focus on validating its use across AE subtypes and exploring its role in treatment monitoring and individualized care.

## Data Availability

The raw data supporting the conclusions of this article will be made available by the authors, without undue reservation.
